# Sleep quality among undergraduate students of a medical college in Nepal during COVID-19 pandemic: an online survey

**DOI:** 10.12688/f1000research.53904.1

**Published:** 2021-06-28

**Authors:** Dhan Shrestha, Suman Prasad Adhikari, Namrata Rawal, Pravash Budhathoki, Subashchandra Pokharel, Yuvraj Adhikari, Pooja Rokaya, Udit Raut

**Affiliations:** 1 Department of Emergency Medicine, Mangalbare Hospital, Urlabari, 1, 56600, Nepal; 2 Department of Neuro-psychiatry, Nepalese Army Institute of Health Sciences, Shree Birendra Hospital, Kathmandu, 3, 44600, Nepal; 3 Department of Emergency Medicine, Dr Iwamura Memorial Hospital, Bhaktapur, 3, 44800, Nepal; 4 Medical student, Nepalese Army Institute of Health Sciences, Kathmandu, 3, 44600, Nepal

**Keywords:** COVID-19, Medical students, Nepal, Sleep, Sleep Deprivation

## Abstract

Background

Good sleep quality is associated with a diverse range of positive outcomes such as better health, less daytime sleepiness, well-being, and proper psychological functioning. Sleep deprivation or poor sleep quality leads to many metabolic, endocrine, and immune changes. Many studies have shown changes in sleep schedule along with worsening of sleep quality during the COVID-19 pandemic.

Methods

This cross-sectional study was conducted among students of a medical college in Kathmandu, Nepal from January 13, 2021, to February 15, 2021. A stratified random sampling technique was used. Data were collected using the Pittsburgh Sleep Quality Index (PSQI). Questionnaires that were completely filled were included and analyzed using STATA vs. 15.

Results

168 medical students filled out the questionnaires with a response rate of 88.42%. 30.36% (n=51) of respondents were having poor quality of sleep (PSQI total score of > 5) with an average PSQI score of 4.24±2.19. Unadjusted logistic regression analysis showed significantly higher odds of poor quality of sleep among females (OR, 2.25; CI, 1.14-4.43) comparing to male and the relation persists even adjusting with age and year in medical school (aOR, 2.81; CI, 1.35-5.86)  Adjusting with age and gender 4 ^th^-year MBBS students were 82% less likely to have a poor quality of sleep compared to 2 ^nd^-year MBBS students (aOR, 0.18; CI, 0.04-0.76). Poor quality of sleep is common among medical students.

Conclusions

More than a quarter of medical students have a poor quality of sleep as per our study. So, education and awareness should be aroused among medical students regarding the detrimental effects of poor quality sleep on daily activities, physical and mental well-being, and the overall quality of life.

## 1. Introduction

The novel coronavirus (COVID-19) that first appeared in Wuhan has been spreading around the world. ^1^ The World Health Organization declared it a pandemic in March 2020. ^2^ The pandemic has brought not only the fear of infection & death but also unbearable psychological pressure and disturbances. ^3, 4^ Good sleep quality is associated with a diverse range of positive outcomes such as better health, less daytime sleepiness, well-being, and proper psychological functioning. ^5^ Sleep deprivation or poor sleep quality leads to many metabolic, endocrine, and immune changes. ^6^ So, the importance of good quality sleep cannot be stressed more.

Many studies have shown changes in sleep schedule along with worsening of sleep quality during the lockdown. ^7^ Findings regarding sleep duration and sleep quality are mixed during the COVID-19 pandemic. ^7– 13^ Most studies examining differences in sleep quality have shown poorer sleep quality during the pandemic. ^12– 16^ Studies in different parts of the world showed that sleep quality was poorer during the lockdown period relative to the pre-lockdown period. ^7, 10– 12, 14^ Compared to the pre-lockdown period, there was a shift to a later bedtime and waking time, with a reduction in nighttime sleep and an increase in daytime napping during the lockdown period. Also, there was evidence of greater sleep latency and poorer sleep efficiency during the pandemic. ^13, 17^ Especially, undergraduate medical students have poor sleep quality, internet addiction, and depression. ^18– 20^

Sufficient studies have not been done in Nepal regarding the sleep quality of medical students during the COVID-19 pandemic. A study on Nepalese college students has shown the poor quality of sleep among the majority of them. ^21^ Higher prevalence of poor sleep quality among medical students as compared to other non-medical students and the general population has also been reported and several factors including medical students’ attitudes, knowledge of sleep, and academic demands have been identified as the causative factors. ^22^

We want to determine the quality of sleep in medical students during this pandemic. Thus, this study aims to assess the impact of this enormous change caused by the COVID-19 pandemic on the sleep quality of medical students of a medical college in Nepal during the COVID-19 pandemic.

## 2. Methods

### 2.1 Study design and settings

This is a cross-sectional study done among the undergraduate medical students (first to the fourth year) of Nepalese Army Institute of Health Sciences (NAIHS), Kathmandu, Nepal from January 13, 2021, to February 15, 2021. The Institutional Review Committee (IRC) of NAIHS approved the study (Reference no: 374). At the time of data collection, all the participants were informed about the study and its objectives. Consent was taken from the participants by incorporating the consent form in the questionnaire itself. So, all the participants are understood to have given consent (details of questionnaire and consent form attached as **extended data**).

The study participants were not recruited as front-liners to tackle the COVID-19 pandemic during or before the study period. All of them were restricted in their homes during the lockdown period. None of the participants had been infected by COVID-19 up until the study period. The stratified random sampling method was used. Data were collected from the participants after receiving their consent, via Google forms sent out by email explaining the objective of the study in the form itself. The participation was voluntary and anonymity was assured to the participants. The participants didn’t receive any incentives.

### 2.2 Study sample

The sample size for the study was calculated using the Cochrane formula. Total students from 1st to 4th year in NAIHS are 423. The calculated sample size was 169. Considering and adding 10% as a non-response rate, the final sample size of 186. The questionnaire was emailed to 190 participants, among which only 168 responded giving a response rate of 88.42%. Detail of sample size calculation is as follow:

Sampling method: Stratified random sampling

Sample size: n = Z ^2^*p*q/e ^2^

      = (1.96) ^2^*0.76*(1-0.76)/0.05 ^2^

      = 280.28

      = 280

where,

n = calculated sample size

Z = 1.96 at 95% Confidence Interval

p = prevalence of poor sleep quality taken from previous study (76%) 10

q = 1-p

e = Margin of error (5%)

Total students from 1 ^st^ to 4 ^th^ year in NAIHS-COM (N) = 423

Adjusted sample size (n′) = n/ [1+ {(n − 1)/N}]

          = 280/ [1+ {(280 − 1)/423}]

          = 168.72

          = 169

Considering and adding 10% as a non-response rate, our final sample size = 186.

We sent the questionnaire to 190 participants.

Participants were selected using stratified random sampling in such a way that every student from first to fourth has an equal chance of being selected. Firstly, a name list of students of the first to the fourth year was obtained from the administration of the institute, and each student was assigned a particular random number. An equal proportion of the male and female students from each year were selected for the study. Since the total number of students in each year was different, the total number of participants was different in a different year. Then the study participants were selected randomly using the computer random number generator maintaining equal proportion of students from each year and equal proportion of males and females in each year [(first year: 45; female = 15, male = 30); (second year: 45; female = 13, male = 32); (third year: 50; female = 17, male = 33); (fourth year: 50; female = 15, male = 35)].

We emailed the questionnaire to the participants as everyone was at their homes because of the COVID-19 pandemic imposed lockdown.

### 2.3 Study instrument

We used the standard and validated Pittsburgh Sleep Quality Index (PSQI), which was developed by researchers at the University of Pittsburgh in 1988 AD. The questionnaire included baseline variables like age, sex, academic year, and questions addressing participants’ sleep habits and quality i.e. PSQI. The PSQI assesses the sleep quality during the previous month and contains 19 self-rated questions that yield seven components: subjective sleep quality sleep, latency, sleep duration, sleep efficiency and sleep disturbance, and daytime dysfunction. Each component is to be assigned a scored that ranges from zero to three, yielding a PSQI score in a range that goes from 0 to 21. A total score of 0 to 4 is considered as normal sleep quality; whereas, scores greater than 4 are categorized as poor sleep quality. ^23^ We typed the questionnaire in the Google form and sent them by email to the randomly selected study participants.

### 2.4 Statistical methods

Data collected from students through the Google forms were extracted to Google sheets, cleaned in Excel, and then imported and analyzed using STATA software v.15. Simple descriptive analysis was performed to see the response for every PSQI variable. Then calculation performed following PSQI form administration instructions (attached in **the extended data**). We ran logistic regression analysis taking PSQI score-based category as the dependent variable and age, gender, and years in medical school as the independent variable. Poor quality of sleep was the outcome of interest so logistic regression analysis was run for the occurrence of poor quality of sleep to good quality of sleep. Firstly binary logistic regression analysis ran across the quality of sleep to gender, age, and year in medical school to estimate the unadjusted odds ratio (OR). Then multiple logistic regression ran to estimate adjusted OR across the quality of sleep to gender, age, and year in medical school. For logistic regression purposes, PSQI score-based quality of sleep was labeled as zero for good quality of sleep and one as poor quality of sleep, and odds of occurrence of poor quality of sleep to good quality of sleep estimated.

## 3. Results

Among the mailed 190 individuals from 1 ^st^ to 4 ^th^ year of a medical school, 168 only filled the Google form making the response rate of 88.42%. The majority (n = 108, 64.29%) were male with a mean age of 21.57 ± 1.52 years. The majority (n = 110, 65.48%) of students were staying single while the rest used to share their room. The average sleep hour in the last month was 7:27:20.09 ± 1:25:49.79 hour and sleep latency was 24.92 ± 25.97 minutes. 30.36% (n = 51) of respondents were having poor quality of sleep (PSQI total score of > five) with an average PSQI score of 4.24 ± 2.19 ( Table 1).

**Table 1.** Participant’s basic details and sleep quality.

Variables		Frequency	Percent
Gender	Female	60	35.71
	Male	108	64.29
Age: Mean ± SD = 21.57 ± 1.52 (Median: 22, IQR: 20-23)			
Medical school class	1st year, MBBS	39	23.21
	2nd year, MBBS	38	22.62
	3rd year, MBBS	47	27.98
	4th year, MBBS	44	26.19
Do you have a bed partner or roommate?	No bed partner or roommate	110	65.48
	Partner in the same bed	3	1.79
	Partner in the same room, but not the same bed	52	30.95
	Partner/roommate in other room	3	1.79
During the past month, hours of actual sleep (Mean ± SD): 7:27:20.09 ± 1:25:49.79 (Median: 7:30:00.00)			
During the past month, sleep latency (in minutes) (Mean ± SD): 24.92 ± 25.97 (Median: 15.5)			
PSQI total score (Mean ± SD): 4.24 ± 2.19 (Median:4)			
PSQI category (sum score < 5 or > 5)	Good sleep quality	117	69.64
	Poor sleep quality	51	30.36

Specific questions on trouble sleeping in last month showed 24(14.3%) were having trouble getting sleep within 30 minutes three or more times a week. Similarly, 12(7.1%) mentioned they wake up in the middle of the night or early morning three or more times a week. Majority i.e. 44% did not use to get up to use the bathroom during the past month. Likewise, only 1.2% and 1.8% had trouble sleeping because they could not breathe comfortably and cough or snore loudly respectively for three or more times a week. When questioned if they had difficulty sleeping because of too cold or too hot feeling three or more times a week, 4.8% and 2.4% responded positively. The majority, 36.9%, and 75.6% did not have trouble sleeping because of bad dreams and having pain respectively during the last month. 98.8% of the participants did not have to take medicine to help him/her sleep during the last month. However, 1.2% had trouble staying awake while driving, eating meals, or engaging in social activities during the past month. Among those who had roommate or bed partner, 6.9%, 1.7%, and 3.4% snored loudly, took long pause between breaths while asleep, and twitched or jerked legs while asleep respectively for three or more times a week during the past month whereas none of them had episodes of disorientation or confusion during sleep ( Table 2).

**Table 2.** Factors affecting sleep in last month at the time of response to the survey.

PSQI scale based questionnaires	Less than once a week	Not during the past month	Once or twice a week	Three or more times a week	Total
5. During the past month, how often have you had trouble sleeping because of you...					
a. Cannot get to sleep within 30 minutes	29(17.3%)	77(45.8%)	38(22.6%)	24(14.3%)	168(100.0%)
b. Wake up in the middle of the night or early morning	48(28.6%)	75(44.6%)	33(19.6%)	12(7.1%)	168(100.0%)
c. Have to get up to use the bathroom	36(21.4%)	74(44.0%)	47(28.0%)	11(6.5%)	168(100.0%)
d. Cannot breathe comfortably	19(11.3%)	141(83.9%)	6(3.6%)	2(1.2%)	168(100.0%)
e. Cough or snore loudly	20(11.9%)	139(82.7%)	6(3.6%)	3(1.8%)	168(100.0%)
f. Feel too cold	41(24.4%)	95(56.5%)	24(14.3%)	8(4.8%)	168(100.0%)
g. Feel too hot	27(16.1%)	127(75.6%)	10(6.0%)	4(2.4%)	168(100.0%)
h. Had bad dreams	60(35.7%)	62(36.9%)	31(18.5%)	15(8.9%)	168(100.0%)
i. Have pain	26(15.5%)	127(75.6%)	10(6.0%)	5(3.0%)	168(100.0%)
7. During the past month, how often have you taken medicine to help you sleep (prescribed or "over the counter")?	1(0.6%)	166(98.8%)	1(0.6%)	-	168(100.0%)
8. During the past month, how often have you had trouble staying awake while driving, eating meals, or engaging in the social activity?	18(10.7%)	130(77.4%)	18(10.7%)	2(1.2%)	168(100.0%)
10. If you have a roommate or bed partner, ask him/her how often in the past month you have had...					
a. Loud snoring	9(15.5%)	42(72.4%)	3(5.2%)	4(6.9%)	58(100.0%)
b. Long pauses between breaths while asleep	5(8.6%)	50(86.2%)	2(3.4%)	1(1.7%)	58(100.0%)
c. Legs twitching or jerking while you sleep	8(13.8%)	41(70.7%)	7(12.1%)	2(3.4%)	58(100.0%)
d. Episodes of disorientation or confusion during sleep	4(6.9%)	52(89.7%)	2(3.4%)	-	58(100.0%)

**Figure 1.**
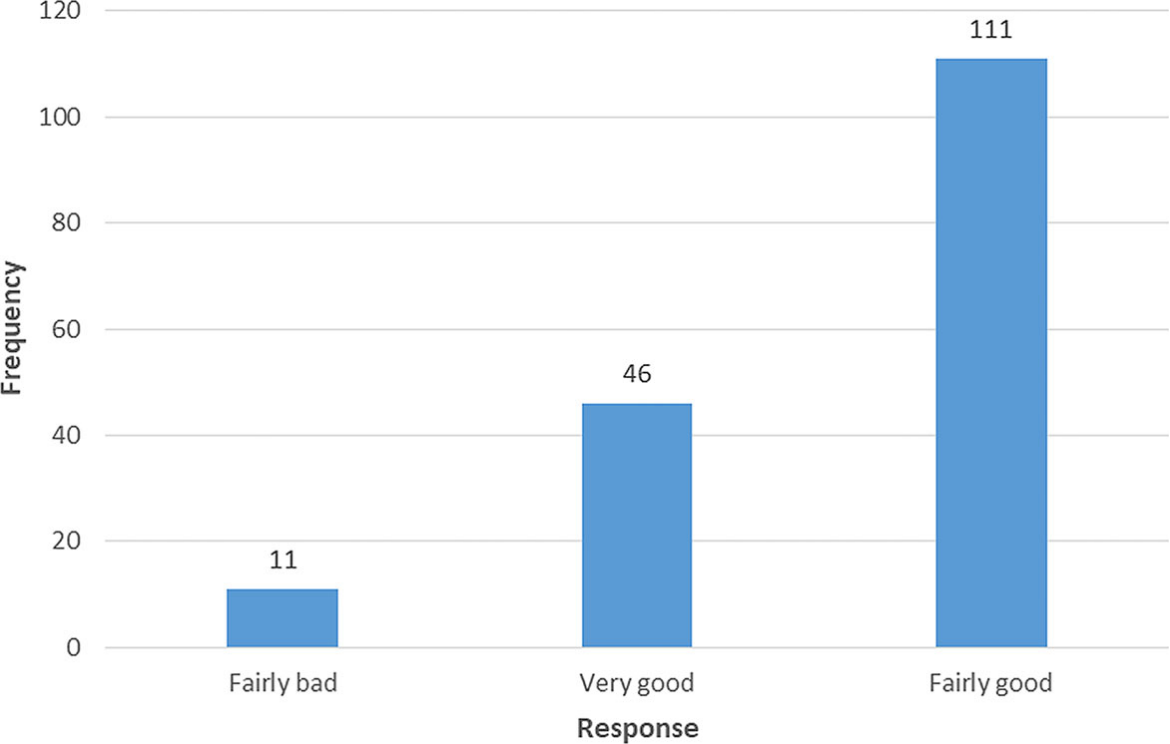
Response on “During the past month, how would you rate your sleep quality overall?”.

Among 168 respondents, 111(66.1%) responded to have a fairly good quality of sleep while asking to rate their overall sleep quality in the last month ( Figure 1).

**Figure 2.**
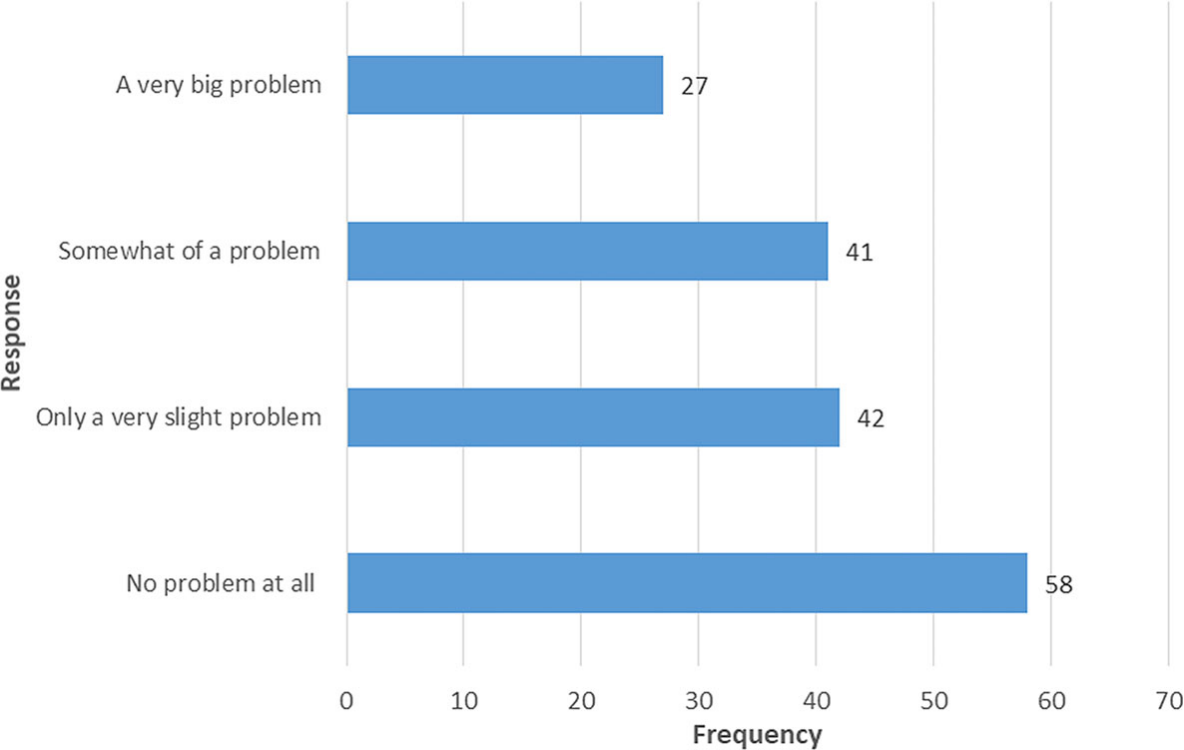
Response on “During the past month, how much of a problem has it been for you to keep up enough enthusiasm to get things done?”.

Fifty-eight (34.5%) responded no problem at all when asking for a problem to keep up enough enthusiasm to get things done during the past month, while 27 (16.1%) responded a very big problem ( Figure 2).

Unadjusted logistic regression analysis showed significantly higher odds of poor quality of sleep among females (OR, 2.25; CI, 1.14-4.43) comparing to male and the relation persisted even adjusting with age and year in medical school (aOR, 2.81; CI, 1.35-5.86) ( Table 3). There was no significant difference in the quality of sleep across years in medical school while running an unadjusted logistic regression analysis. But, adjusting with age and gender 4 ^th^-year MBBS students are 82% lesser likely to have a poor quality of sleep comparing with 2 ^nd^-year MBBS students (aOR, 0.18; CI, 0.04-0.76) ( Table 3).

**Table 3.** Binary and multiple logistic regression taking age, gender, and years in medical school for PSQI score.

Variables		Unadjusted				Adjusted			
		OR	[95% Conf. Interval]		p-value	OR	[95% Conf. Interval]		p-value
Years in Medical school	1st year, MBBS ^®^								
	2nd year, MBBS	1.04	.4043604	2.674842	0.935	.7708897	.2731356	2.175736	0.623
	3rd year, MBBS	1.133333	.4640643	2.767816	0.784	.5756672	.174416	1.900014	0.365
	4th year, MBBS	.4444445	.1610749	1.226329	0.117	.1766567	.0412005	.7574558	0.020*
Age		.9777632	.7871435	1.214545	0.839	1.361479	.9609949	1.928861	0.083
Gender	Male ^®^								
	Female	2.252747	1.144979	4.432282	0.019*	2.811422	1.348437	5.86167	0.006*

Note ®: Reference category, 95% Confidence interval, and 5% standard error considered with p-value or <0.05 as a level of significance. * Significant result. PSQI score based category for sleep was assigned as 0/1 [good quality of sleep (0) to poor quality of sleep (1)].

## 4. Discussion

The COVID-19 pandemic in 2020 has imposed a substantial effect on psychological, health, and social issues all over the world. Medical students, like everyone else, have also suffered from these issues. Medical education is based more on a practical approach rather than just a theoretical core. Therefore, classes shifting from the classroom to online platforms have, without any doubt; affected medical education more than anything else. However, the impact of the pandemic on the psychological well-being and sleep quality of medical students has not been assessed and addressed to a good extent in Nepal. Therefore, this study uses the PSQI to determine the quality of sleep among the medical students of a medical college in Kathmandu, Nepal during the COVID-19 pandemic.

Our study showed that 30.36% of medical students had poor quality of sleep (PSQI global score >5) but a study has done just before the pandemic in the Nepalese medical students showed that 44.23% of students had poor sleep quality. ^24^ A study from India during the COVID-19 pandemic had 34.6% of medical students with poor sleep quality which was in line with the result of our study. ^25^ Previous studies from Pakistan on medical students in 2013 and Nepal on undergraduate students in 2015 showed 39.5% and 35.4% poor quality sleepers respectively. ^19, 26^ Our findings were in contrast to a study in China which showed a high prevalence of sleep disorders among adolescents and young adult students due to the stress and anxiety caused by the pandemic. ^27^ Previous studies in the Nepalese general population and health care workers had found a low level of psychological distress due to the COVID-19 pandemic in Nepal which may explain better sleep quality among medical students in Nepal. ^28, 29^

One of the positive findings of this study is that none of the participants had to take medication to help them sleep whereas 10.2% of Saudi Arabian physicians used sleeping pills once or twice a week during the COVID-19 pandemic. ^30^ 14.3% of our study participants had trouble falling asleep within 30 minutes thrice or more in a week, which was less compared to 24.7% in Saudi Arabian physicians during the COVID-19 pandemic. ^30^ Our study showed significantly higher odds of poor quality sleep among female students (OR, 2.25; CI, 1.14-4.43). A study on Pakistani medical students in 2013 also showed that more females had poor quality sleep i.e. 44% as compared to 32.8% male poor sleepers. ^26^ Similarly, Goweda RA *et al*. also found that sleep disorders were more common among female medical students based on a study done in Saudi Arabia during the COVID-19 pandemic. ^31^ Increased incidence of poor sleep quality among females is justifiable as sleep disorder symptoms are usually greater in women as compared to men. ^28, 32^ So, more focus should be shed upon female students when programs are formulated to improve the sleep quality among medical students. Sleep quality among students across years in medical school was not significantly different while running unadjusted logistic regression analysis in the case of our study. Studies from Brazil and Saudi Arabia assessed the sleep quality of first and second-year medical students to be poor as compared to other years. ^30, 33^ We found that the fourth-year medical students were less likely to have a poor quality of sleep compared to second-year students which may be due to greater academic experience, and exposures during higher academic classes. In contrast, a previous study in China found that senior high school students had greater sleep problems due to increased academic burden and difficulty compared to junior school students. ^27^

Though our study shows less number of poor quality sleepers as compared to other studies discussed, ^19, 24, 25^ 30.36% is still a big number. We cannot stay still and satisfied based on the result of this study and the concerned authorities should try their best on bringing this number down to as minimum as possible. Because, poor quality sleep, without a doubt, will have a long-term impact on the mental well-being of the students. And no country would want future doctors who are not mentally healthy.

The number of students with poor sleep quality might decrease after the fear and restrictions because COVID-19 subsides and life will return to normality. Alternatively, the number might go up, as the students return to their hectic schedules of classes, clinical postings, and a lot of studying. Since the scenario post-COVID is still unpredictable, frequent monitoring of sleep health and habits in medical students for raising awareness about sleep quality and problems should be planned and implemented.

Our study is not without limitations. We used a self-reporting questionnaire. Therefore, information bias is a major risk for this study. Our study was conducted among the students of a single institution. Therefore, the results might not be extrapolated to all the medical colleges of Nepal. Likewise, recall bias and subjectivity bias could have also affected the result of our study. Despite the limitations and biases, this study will surely provide a reference for further researches in this particular field.

## 5. Conclusions

Poor quality of sleep is prevalent in 30.36% of medical students of a medical college in Nepal during the COVID-19 pandemic. Further studies have to be done to delve deeper into the sleep habits and problems of the medical students to determine the risk factors and causation of poor sleep quality. Furthermore, awareness should be aroused about the importance of proper amount and quality of sleep as well as detrimental effects of poor quality sleep on daily activities, physical and mental well-being, and the overall quality of life.

## Data availability

### Underlying data

Figshare. Sleep quality among undergraduate students of a medical college in Nepal during COVID-19 pandemic: an online survey. DOI: https://doi.org/10.6084/m9.figshare.14770182.v2. ^34^

This project contains the following underlying data:

- We used the standard and validated Pittsburgh Sleep Quality Index (PSQI), which was developed by researchers at the University of Pittsburgh in 1988 AD. The questionnaire included baseline variables like age, sex, academic year, and questions addressing participants’ sleep habits and quality i.e. PSQI. The PSQI assesses the sleep quality during the previous month and contains 19 self-rated questions that yield seven components: subjective sleep quality sleep, latency, sleep duration, sleep efficiency and sleep disturbance, and daytime dysfunction. Each component is to be assigned a scored that ranges from zero to three, yielding a PSQI score in a range that goes from 0 to 21. A total score of 0 to 4 is considered as normal sleep quality; whereas, scores greater than 4 are categorized as poor sleep quality.
- Data collected from students through the Google forms were extracted to Google sheets, cleaned in Excel, and then imported and analyzed using STATA 15. Simple descriptive analysis was performed to see the response for every PSQI variable. Then calculation performed following PSQI form administration instructions.

Data are available under the terms of the Creative Commons Zero “No rights reserved” data waiver (CC BY 4.0 Public domain dedication).

### Extended data

Figshare. Sleep quality among undergraduate students of a medical college in Nepal during COVID-19 pandemic: an online survey. DOI: https://doi.org/10.6084/m9.figshare.14770182.v2. ^34^

Data are available under the terms of the Creative Commons Zero “No rights reserved” data waiver (CC BY 4.0 Public domain dedication).

## Authors’ contributions

DBS, SPA, NR, PB, SP, YA, PR, and UR contributed to the concept and design, methodology, and data collection. DBS contributed to the analysis, and interpretation of data. DBS, PB, SP, YA, PR, and UR contributed to the literature search, and initial manuscript drafting. SPA, NR involved in the revision and intellectual interpretation of the manuscript.

All authors were involved in drafting and revising the manuscript and approved the final version.
