# Supplementary material for: Sleep quality among undergraduate students of a medical college in Nepal during COVID-19 pandemic: an online survey
Source: F1000Res. 2021 Jul 21;10:505. Originally published 2021 Jun 28. [Version 2] doi: 10.12688/f1000research.53904.2 (PMC8261762; doi:10.12688/f1000research.53904.2)
Supplement: Peer review history

# Peer review: Reviewer response for version 1

**DOI:** 10.5256/f1000research.57334.r88342

**Published:** 2021-07-06

**Authors:** Samata Nepal

**Affiliations:** 1 Department of Community Medicine, Lumbini Medical College Teaching Hospital, Palpa, Nepal

**Abstract:**

- Please use punctuation wherever required, e.g. "well-being and..." instead of "well-being, and...".
- "from January 13, 2021 to February..." instead of "from January 13, 2021, to February...".
- The sentence starting with a number is not appropriate in the Results section, second sentence. Please consider "Around 30% (n=51) of respondents..." or "Thirty percent (n=51) of respondents...".

**Introduction:**

- Paragraph 2, line 5: "Compared to the pre-lockdown...", this sentence needs to be paraphrased. Consider "When compared to pre-lockdown period, there was a later shift to bedtime and waking time, which reduced sleep during the night and increased daytime napping".
- Paragraph 4, line 1: The first sentence is not needed, please consider "This study aims..."

**Methods:**

- Paragraph 1, line 2: Consider "January 13, 2021 to February 15".
- Paragraph 3, line 3: "The questionnaire was...", please remove this sentence as the same sentence is repeated in the next paragraph.
- The last line of 2.2 is not required. "We emailed the..." has been mentioned already.
- 2.3 Study instrument, line 5: Please consider "component is assigned score...".
- 2.3 Study instrument, line 7: Please consider "The questionnaire was typed in the Google form and...". The use of passive format is better.
- Please mention clearly if the questionnaire was emailed or the link of the Google form was emailed. Please consider using uniformity throughout the Methods section.
- 2.4 Statistical methods: line 1: "Google forms was extracted...".
- 2.4 Statistical methods, line 5: Please consider "Logistic regression analysis was done...". The use of passive format is better.
- Repeated use of the word "ran" in 2.4 can be avoided, e.g. "logistic regression analysis was run...", "multiple logistic regression ran...".
- Please consider uniformity in writing either "good/poor quality sleep" or "good/poor quality of sleep".

**Results:**

- Table 1: The table with the variable "Do you have a bed partner or roommate?" - the last row has a sub-variable: " Partner/roommate in another room". Please clarify this option.

Is the work clearly and accurately presented and does it cite the current literature?

Yes

If applicable, is the statistical analysis and its interpretation appropriate?

Yes

Are all the source data underlying the results available to ensure full reproducibility?

Yes

Is the study design appropriate and is the work technically sound?

Yes

Are the conclusions drawn adequately supported by the results?

Yes

Are sufficient details of methods and analysis provided to allow replication by others?

Yes

Reviewer Expertise:

Public health

I confirm that I have read this submission and believe that I have an appropriate level of expertise to confirm that it is of an acceptable scientific standard.

# Peer review: Reviewer response for version 1

**DOI:** 10.5256/f1000research.57334.r88340

**Published:** 2021-07-01

**Authors:** Roshan Acharya

**Affiliations:** 1 Department of Internal Medicine, Cape Fear Valley Medical Center, Fayetteville, NC, USA

This study addresses one of the important problems prevalent among medical students. It has been conducted following the proper research methods. The title of the study is appropriate. The manuscript seems to be technically sound. The statistical analysis has been done appropriately. All the data underlying the findings are also made fully available. The conclusion includes the results, and recommendations are given accordingly. The paper seems to be well-written despite a few minor errors.

 My comments regarding the paper are as follows:

 Major correction: None

 Minor corrections:

**Abstract**:

- *“Unadjusted logistic regression analysis showed significantly higher odds of poor quality of sleep among females (OR, 2.25; CI,1.14-4.43) comparing to male and the relation persists even adjusting with age and year in medical school (aOR, 2.81; CI, 1.35-5.86)”*

Please put a full stop(.) after this sentence.

  

- *“So, education and awareness should be aroused among medical students regarding the detrimental effects of poor quality sleep on daily activities, physical and mental well-being, and the overall quality of life.”*

In this sentence, the replacement of the word ‘aroused’ with ‘raised’ would be better.

**Introduction**:

- *“Studies in different parts of the world showed that sleep quality was poorer during the lockdown period relative to the pre-lockdown period.7,10–12,14 Compared to the pre-lockdown period, there was a shift to a later bedtime and waking time, with a reduction in nighttime sleep and an increase in daytime napping during the lockdown period. Also, there was evidence of greater sleep latency and poorer sleep efficiency during the pandemic.”*

In the second paragraph, you have used past tense. I think it would be better to use present tense instead.

**Methods: **

- ***“*** *The stratified random sampling method was used.”*

This statement has been mentioned thrice in the methods section itself, once in **Study design and settings**, twice in **Study sample**. Please mention it only once, wherever you think is suitable.

**Study sample:**

- *“Detail of sample size calculation is as follow:”*

Here ‘as follows’ is the correct one, please correct it.

**Study instrument:** 

- “ *Each component is to be assigned a scored that ranges from zero to three, yielding a PSQI score in a range that goes from 0 to 21.” *

In this sentence, correct ‘assigned a scored’ to ‘assigned a score’

**Discussion**:

- *“Our study showed that 30.36% of medical students had poor quality of sleep (PSQI global score >5) but a study has done just before the pandemic in the Nepalese medical students showed that 44.23% of students had poor sleep quality.”*

Here, two points; First, please write ‘study done’ in place of ‘study has done’.

 You have discussed and compared the findings of the 2nd and the 4th year, but not compared them to other years. Also, there is no discussion on why the score of the 3rd year is the highest. Please address this as well.

 Second: PSQI change it to ‘equal or greater than 5’. Please see my last point for further clarification.

**Conclusion**:

- *“ Furthermore, awareness should be aroused about the importance of proper amount and quality of sleep as well as detrimental effects of poor quality sleep on daily activities, physical and mental well-being, and the overall quality of life.”*

In this sentence, the replacement of the word ‘aroused’ with ‘raised’ would be better.

**Table 1:**

 In the methodology it was mentioned that: A total score of 0 to 4 is considered as normal sleep quality; whereas, scores greater than 4 are categorized as poor sleep quality. But in Table 1, it is otherwise: *PSQI category (sum score < 5 or > 5).* It should be corrected in Table 1 for uniformity.

Is the work clearly and accurately presented and does it cite the current literature?

Yes

If applicable, is the statistical analysis and its interpretation appropriate?

Yes

Are all the source data underlying the results available to ensure full reproducibility?

Yes

Is the study design appropriate and is the work technically sound?

Yes

Are the conclusions drawn adequately supported by the results?

Yes

Are sufficient details of methods and analysis provided to allow replication by others?

Yes

Reviewer Expertise:

Clinical research

I confirm that I have read this submission and believe that I have an appropriate level of expertise to confirm that it is of an acceptable scientific standard.
